# A Lecture on the Determinative Cause of Labor, Delivered to His Class, in Lebanon, Tenn.

**Published:** 1849-08

**Authors:** F. H. Gordon

**Affiliations:** Lebanon, Tenn.


### THE WESTERN JOURNAL OF MEDICINE AND SURGERY.

AUGUST, 1849.

---

ART. I.—*A Lecture on the Determinative Cause of Labor*, delivered to his Class, in Lebanon, Tenn., by F. H. GORDON, M. D.

The long and anxiously looked for "Theoretical and Practical Treatise on Human Parturition," by Professor Miller, has appeared at last. I have not yet had the pleasure of its perusal; but from the review of it in the Western Journal, I take it to be such a work as I expected from so clear a head, and such as will much interest and benefit the profession. I have notes taken some years ago from the able and lucid lectures of Professor M., which contain more important matter, than I ever saw in the same space. His arguments generally, and especially upon controverted points, were so clear and forcible, that he seldom failed to convince me of the truth of his positions.

But independence of thought will sometimes lead the mind to differ from the highest authority; and it is my lot to view differently from Prof. M. one important subject of his book; I mean "the determinative cause of labor." And I know he will fully appreciate the reasons I may advance against his position. He contends that the determinative cause of labor, is "irritation of the cervix and especially of the os uteri, arising from the contact of the ovum;" or, as I heard him briefly express it in a lecture, it is the "sensible impression of the fetus upon the cervix and os uteri."

The arguments in support of this position, are 1st. The unfolding of the neck of the womb so as to allow contact of the ovum, is deferred till a short time before labor sets in. 2d. The analogy of the urinary bladder and the rectum being excited to expulsive efforts, by the contact of their contents. 3d. The uterus can be excited to expulsive efforts at any time, by irritation of its orifice.

The first and third are statements of facts, which must be admitted; and the second position presents a striking analogy. But I think these facts and this analogy are but *presumptive* evidences of the truth of the position, and not conclusive proof. There is an adverse position supported, in my judgment, by evidences more conclusive. To be clearly understood let it be plainly stated.—The determinative cause of labor, is *an increase of vital force given to the womb at term.* I do not deny—nay, I believe it—that when the womb is fully supplied with sensibility and vital force for its task at term, contact of the ovum may call this force into action; but my position is, that in a healthy uterus, contact is not adequate to cause expulsive efforts during the course of gestation; because vitality is not so abundant as to give the organ the requisite excitability and power before term. True, the finger in the os uteri, or the hand grasping the womb, or a fall, may lash the organ into fury at any stage; but this is violence or disease—not ordinary stimulus and ordinary excitability, or the uterus would not take on abnormal action.

Prof. M.'s first argument, that the neck of the uterus is not unfolded till a late period of pregnancy, is introduced to prove that this is Nature's provision against abortion, or premature delivery; and also her plan of bringing on labor at term, by then presenting a sensitive os uteri to the impression of the ovum. But abortions and premature labors do take place before term and before the neck is unfolded. Is labor now the effect of "sensible impression?" This is regarded as the result of disease or violence. So it is; but it proves 1st. That labor can exist without contact of the ovum with the unfolded neck; 2d. That moderate vitality of the womb may be called into powerful action by a powerful stimulus; and 3d. That morbid excitability of the organ may empower it to be excited into violent action by ordinary stimulus.

In first pregnancies, the neck unfolds more slowly than in subsequent ones, and yet in the first instance women are less apt to go to the full term of 40 weeks. I see no way to account for this unless by supposing that vitality is earlier supplied in the first instance.

Again. If the womb is, at all stages, possessed of the same amount of vitality, and labor is induced by contact of the fetus with the neck, we should expect all large children to be born before full term; because a large child might produce as much effect on a half unfolded neck, as a small one on a fully unfolded neck. But the first child is apt to be less than subsequent ones, and yet more apt to be born before full term.

From these considerations, it appears that "the office of the cervix uteri is (truly) to retain the ovum till the proper time arrives for its expulsion," and nothing more; and that the determinative cause of labor includes something more than "sensible impression."

But, in support of the position we assume, we need but state as a law of the organization, that whatever an organ is required to do, it is qualified to perform, by being furnished with *an adequate supply of vital force, at the time the requisition is made:* and the converse, vital force is withheld from an organ when it is required to be inert. Need we adduce proof to establish this as a vital law? Its mere statement must demand the assent of most minds. But take a few of the multiplied facts which illustrate the law. From infancy to adult age, the assimilative organs are more highly vitalized, and therefore more active than the depurating organs; hence the infant grows to the stature of puberty. During the meridian of life, vitality is equally distributed to the assimilating and depurating organs, and therefore the individual is stationary in size. But in the decline of life, the depurative organs are more vigorous, and the being diminishes in stature to the end of life.

Various parts of the physical system undergo changes in obedience to the same law of vitalization. At one age, the infant teeth are developed; at another, the permanent teeth come out. The ovaria and mammary glands are rapidly vitalized at puberty. They are speedily developed, and take on exquisite sensibility. At this period, the external genitals are enlarged, and made more sensitive, while the mons veneris, from a new vital action, is soon covered with hair. During these changes the uterus is abundantly vitalized, and takes on the new function of menstruation, the common evidence of its being capable of another vital process, that of propagating the species. After conception, the vital fluid is still more abundantly furnished to all the organs about to develop and rear the new being. They all enlarge still more, become exquisitely sensitive, until, at term, the uterus has vital force enough to expel the fetus into the world, and in a few days the mammary glands are so highly vitalized as to secrete an abundance of milk.

In the decline of life, the catamenia ceases, the ovaries, mammæ, and uterus become atrophied, and the procreative power ceases, because the vital force is withheld.

In the male, similar changes take place, in obedience to the same vital law.

Of the inferior animals, similar remarks may be made. The feathers of fowls, the comb and spurs of cocks, the fangs of serpents, the tusks of boars, and the horns of various animals, are all developed in their due stages of life, and without violating the law regulating the distribution of vital force, none of them could be developed before their due season.

We might pursue this view, and notice the various changes which take place, not only in other animals of a high grade, but in insects, and the vegetable kingdom.—All of these have their rounds of existence, and are furnished with vital force in their different organs *pari passu* with the developments and functions which take place in them.

When the gravid uterus is viewed in relation to this law, and when it is seen that its ordinary course is not to make expulsive efforts till full term, we are compelled to infer, that the excitability and power requisite to expulsion are withheld until the end of gestation. It would be but waste of vital force, to supply the womb with more than its normal functions necessarily expend; and expulsive efforts before term, must be regarded as abnormal, and consequently without that abundant power usually supplied for parturition.

At any stage of gestation, give the uterus the amount of vital fluid which imparts exquisite sensibility and great power, and you at once bring on labor from the presentation of the slightest stimulus.

Were Nature so careless and so prodigal of vital force, as to furnish it freely and equally from conception to the end of parturition, nothing but abortion and misrule could follow. A slight cause, as irritable bowels, an awkward step, vomiting, a jolt in a carriage, laughter, and thousands of little incidents, such as almost daily occur, could not fail to cause abortion. No fetus could mature, and the species would become extinct.

What limits the term of gestation, but the law governing the distribution of vital force? Is it not by this law, that Nature controls all the procreative functions, from the irruption to the decline of the catamenia? And if there is any uniform order, or time for their performance, does this not imply that vital force is furnished to each organ *when* it is required to act, and in an amount adequate to the task required? And the converse, the efficient vital force is withheld till "Nature's own good time," and therefore in the normal course of things, the act can not be accomplished before that time.

According to this view, the reason is plain, why the gravid uterus has its term of gestation—because the requisite sensibility and contractile force are withheld till term, and it is therefore impossible for labor to come on before that time, because the power is wanting. There is not the ability, and therefore the act cannot be done.—But if labor come on before term, it must be in violation of this law. Disease or violence may pervert the law and do mischief; and perversion is a pre-requisite to the mischief.

On the contrary, when gestation has completed its round, and fulfilled all its objects, and the fetus is completely viable without the mother, the womb becomes exquisitely sensitive, and it is now as impossible for it to refrain from expulsive efforts, as before it was impossible for it to make them. Hence the infant is born, because the uterus has the power and can not avoid exerting it. The task is a great one, and the womb is now furnished with a large amount of vital force. The hand placed in the womb during its action, will satisfy any one of its extraordinary power.

I have already admitted that stimulus is an *exciting cause* of labor. That the contact of the ovum prompts the uterus to act, I think probable : but its presence could not call the organ into action, until furnished with (I may call it) the parturient vital fluid, which endows it with exquisite sensibility and great power. The tendency of an organ to act, and the power with which it is capable of acting, depend upon the sum of its vitality and the stimulus conjoined. But the stimulus (i. e. the fetus) is always present, from the beginning to the end of pregnancy ; ergo, the parturient vitality is not furnished till term, because labor is not induced till then.

In further support of this position, it may be remarked that the uterus may be so highly vitalized, as to take on efficient parturient action without the presence of a fetus. This is evinced, when the organ is distended with some morbid product, as gas, hydatids, &c., or in cases of extra-uterine pregnancy. In these cases, it must be allowed, that the uterus takes on expulsive efforts at term, without " sensible impression."

And we may add, that if labor is effected, or altogether caused by the mechanical impression of the fetus, we must be compelled to ascribe the gradual expansion of the cervix uteri, in latter months, to a similar cause, that is, to the distention of the womb, caused by the growth of the fetus. But to this, we would urge the same objections we have before adduced. The neck is obliterated no sooner by large, than by small children. The expansion of the neck, must be in obedience to the same law, which protrudes the horns of quadrupeds, and accomplishes many other objects in their due course of time. But we might as justly ascribe the expansion of body of the womb to the fetus as that of the neck. And by the same parity of reason, we might account for the absolute growth of the uterus, from one or two ounces to three or four pounds. In truth, none of these are done by the fetus, but for it; just as delivery is not accomplished *by* the fetus, but *for* it. All the changes which take place in the mother, from conception to the end of lactation, are *for* the new being—that is, they are accomplished *by* the laws of organization, *for* the perpetuation of the species. The great Author of nature, has wisely not left the propagation of the races of beings to any contingency or important agency of the fetus; but he has established physical laws, by which the end is much more certainly accomplished.

Lebanon, Tenn., April, 1849.

